# Heading for the hills? Evaluating spatial distribution of woodland caribou in response to a growing anthropogenic disturbance footprint

**DOI:** 10.1002/ece3.2362

**Published:** 2016-08-18

**Authors:** Doug MacNearney, Karine Pigeon, Gordon Stenhouse, Wiebe Nijland, Nicholas C. Coops, Laura Finnegan

**Affiliations:** ^1^ fRI Research Caribou ProgramHintonAlbertaCanada; ^2^ fRI Research Grizzly Bear ProgramHintonAlbertaCanada; ^3^ Department of Forest Resources ManagementFaculty of ForestryUniversity of British ColumbiaVancouverBritish ColumbiaCanada

**Keywords:** Philopatry, range fidelity, range shift, spatial ecology, UD overlap, utilization distribution overlap index, winter severity

## Abstract

Anthropogenic landscape change (i.e., disturbance) is recognized as an important factor in the decline and extirpation of wildlife populations. Understanding and monitoring the relationship between wildlife distribution and disturbance is necessary for effective conservation planning. Many studies consider disturbance as a covariate explaining wildlife behavior. However, we propose that there are several advantages to considering the spatial relationship between disturbance and wildlife directly using utilization distributions (UDs), including objective assessment of the spatially explicit overlap between wildlife and disturbance, and the ability to track trends in this relationship over time. Here, we examined how central mountain woodland caribou (*Rangifer tarandus caribou*) distribution changed over time in relation to (i) anthropogenic disturbance, baseline range (defined using telemetry data from 1998 to 2005), and alpine habitat; and (ii) interannual climate variation (North Pacific Index; NPI). We developed seasonal UDs for caribou in west‐central Alberta and east‐central British Columbia, Canada, monitored with GPS collars between 1998 and 2013. We mapped the cumulative annual density of disturbance features within caribou range and used indices of overlap to determine the spatial relationship and trend between caribou UDs, anthropogenic disturbance, baseline range, alpine habitat, and the NPI. Anthropogenic disturbance increased over time, but the overlap between caribou UDs and disturbance did not. Caribou use of alpine habitat during spring, fall, and late winter increased over time, concurrent with a decrease in use of baseline range. Overlap between caribou UDs and disturbance increased during spring and fall following relatively cold, snowy winters (high NPI), but overall, climate did not explain changes in caribou distribution over time. We provide evidence supporting the hypothesis that caribou populations adjust their spatial distribution in relation to anthropogenic landscape change. Our findings could have implications for population persistence if distributional shifts result in greater use of alpine habitat during winter. Monitoring long‐term changes in the distribution of populations is a valuable component of conservation planning for species at risk in disturbed landscapes.

## Introduction

Landscape change from anthropogenic disturbance is recognized as an important factor in the decline of species worldwide (Vors et al. 2007). Understanding the response of wildlife populations to disturbance can help mitigate the impacts of anthropogenic activities (Panzacchi et al. 2012), and monitoring the relationship between wildlife distribution and anthropogenic disturbance is necessary for effective conservation planning (Festa‐Bianchet et al. 2011). Several studies have shown that ungulates use lower quality habitat to avoid anthropogenic disturbance, resulting in an eventual fitness consequence (i.e., reproduction, nutrition, survival; Sawyer et al. 2006; Johnson et al. 2015). Alteration in space use by wildlife populations can be linked to the spatial distribution of disturbances and habitat heterogeneity (Guisan et al. 2013; Johnson et al. 2015), which is valuable in conservation settings where recovery actions need to be prioritized (McDonald‐Madden et al. 2010; Guisan et al. 2013).

The increasing availability of extensive geographic positioning system (GPS) telemetry datasets has resulted in a proliferation of analytical tools for spatial ecologists (Cameron et al. 2005; Sawyer et al. 2006; Clapp and Beck 2015). Spatial distribution is relatively easy to estimate in the form of utilization distributions (UDs) from the kernel density of telemetry data (Worton 1989; Millspaugh et al. 2006), and changes in distribution can be assessed using empirical methods to quantify spatiotemporal overlap between individuals and populations (Fieberg and Kochanny 2005; Keating and Cherry 2009). However, studies that employ the overlap of UDs to assess changes in animal distribution typically consider only the overlap between species or individuals, with the effect of anthropogenic disturbance included as a covariate (e.g., Benson and Patterson 2013; López‐López et al. 2014 but see Millspaugh et al. 2000). Using woodland caribou (*Rangifer tarandus caribou*) as a case study, we propose that the density of anthropogenic disturbance is analogous to a UD and that by quantifying indices of caribou overlap with disturbance using a UD approach, the relationship between caribou UDs and anthropogenic disturbance can be considered directly.

Declines in woodland caribou are believed to be a result of anthropogenic factors leading to habitat fragmentation and loss (Festa‐Bianchet et al. 2011; Hervieux et al. 2013), while changes in spatial distribution of caribou (i.e., range shift) have been attributed to natural and anthropogenic factors (Schindler et al. 2007; Newton et al. 2015). The ability to move between areas in response to the availability of resources, predation risk, and dynamic environmental conditions can be viewed as an adaptation that allows caribou to persist in fire disturbed landscapes, reduce density‐dependent negative effects by using higher quality alternative ranges away from forage‐depleted areas, make use of seasonally available resources through migration, and reduce predation risk by spacing away from predators (Heard et al. 1996; Briand et al. 2009; McDevitt et al. 2009; Newton et al. 2015). High‐quality alternative ranges may no longer exist in landscapes disturbed by anthropogenic activities because of the number of forestry clear‐cuts, energy extraction infrastructure, and linear features such as roads, pipelines, and seismic lines (Kinley and Apps 2001; Tracz et al. 2010). In landscapes disturbed by anthropogenic activities, there is a great variability in the fidelity of individuals to seasonal ranges (see Faille et al. 2010; Tracz et al. 2010); however, at the population scale, caribou have been shown to shift their distribution into lower quality habitat, possibly to increase short term fitness (survival) at the cost of reduced forage quality, lower reproductive rates, and increased susceptibility to stochastic events (Cameron et al. 2005; Hebblewhite et al. 2010; Johnson et al. 2015). In other populations of ungulates (i.e., elk, *Cervus elaphus*), changes to migratory strategies from fully migratory to partially or nonmigratory may be an adaptation to dynamics in predation risk (Middleton et al. 2013); the trade‐offs associated with this hypothesis have not been investigated for caribou in disturbed landscapes although some populations display a wide spectrum of migratory strategies (McDevitt et al. 2009).

Although density‐dependent range shifts have been documented in migratory caribou herds after peaks in population size (e.g., Ferguson et al. 1998; Newton et al. 2015; Mahoney et al. 2016), boreal and mountain caribou exist in relatively small populations well below the carrying capacity of their range, where the effects of density dependence are likely to be weak and population size is believed to be regulated by top‐down effects (Seip 1992; Wittmer et al. 2005; Courtois and Ouellet 2007). Caribou distribution is also dependent on climate variability, and weather conditions can affect caribou behavior and the availability and quality of forage (Sharma et al. 2009). Storm severity and frequency is expected to increase due to climate change (Sharma et al. 2009), and range shifts caused by climate variability could have different management implications than those related to anthropogenic disturbance.

Our objective was to assess trends in the distribution of caribou in relation to anthropogenic disturbance and climatic factors. We investigated the change in spatial distribution of two central mountain caribou herds located in west‐central Alberta and east‐central British Columbia, Canada, over time by quantifying the overlap between caribou UDs and the density and distribution of anthropogenic disturbance features. We conducted our analysis according to caribou seasons to account for seasonal dynamics in life history requirements and proximity to disturbance (Saher and Schmiegelow 2005; Rudolph and Drapeau 2012). We also quantified the annual overlap between seasonal UDs, baseline seasonal range defined using telemetry data from 1998 to 2005, and alpine habitat, a spatially static habitat class with seasonal variation in resource quality (Barten et al. 2001; Natural Regions Committee 2006). Additionally, we examined behavioral parameters of caribou (habitat use, home range size, and movement rate) for changes over time in relation to disturbance and climate.

A shift in the distribution of caribou was previously documented in our study area (Smith et al. 2000; Hebblewhite et al. 2010; Slater 2013) and was hypothesized to be related to increases in anthropogenic disturbance. However, this hypothesis has not been explicitly investigated. We predicted that: (1) overlap between caribou UDs and disturbance would be consistently low despite increases in the disturbance footprint; (2) overlap between contemporary (2006–2013) caribou UDs and baseline (1998–2005) range would decrease over time; and (3) overlap between caribou UDs and alpine habitat would increase over time as alpine areas represent a refuge from anthropogenic disturbance where caribou may benefit from lower predation risk despite seasonal fluctuations in climate severity and forage availability. We also investigated an alternate hypothesis that variation in climate would drive changes in the distribution of caribou and predicted that (4) an increase in the overlap between caribou UDs and alpine habitat would be associated with mild winters with shallow snow packs rather than with an increase in anthropogenic disturbance.

## Methods

### Study area

**Figure 1:**
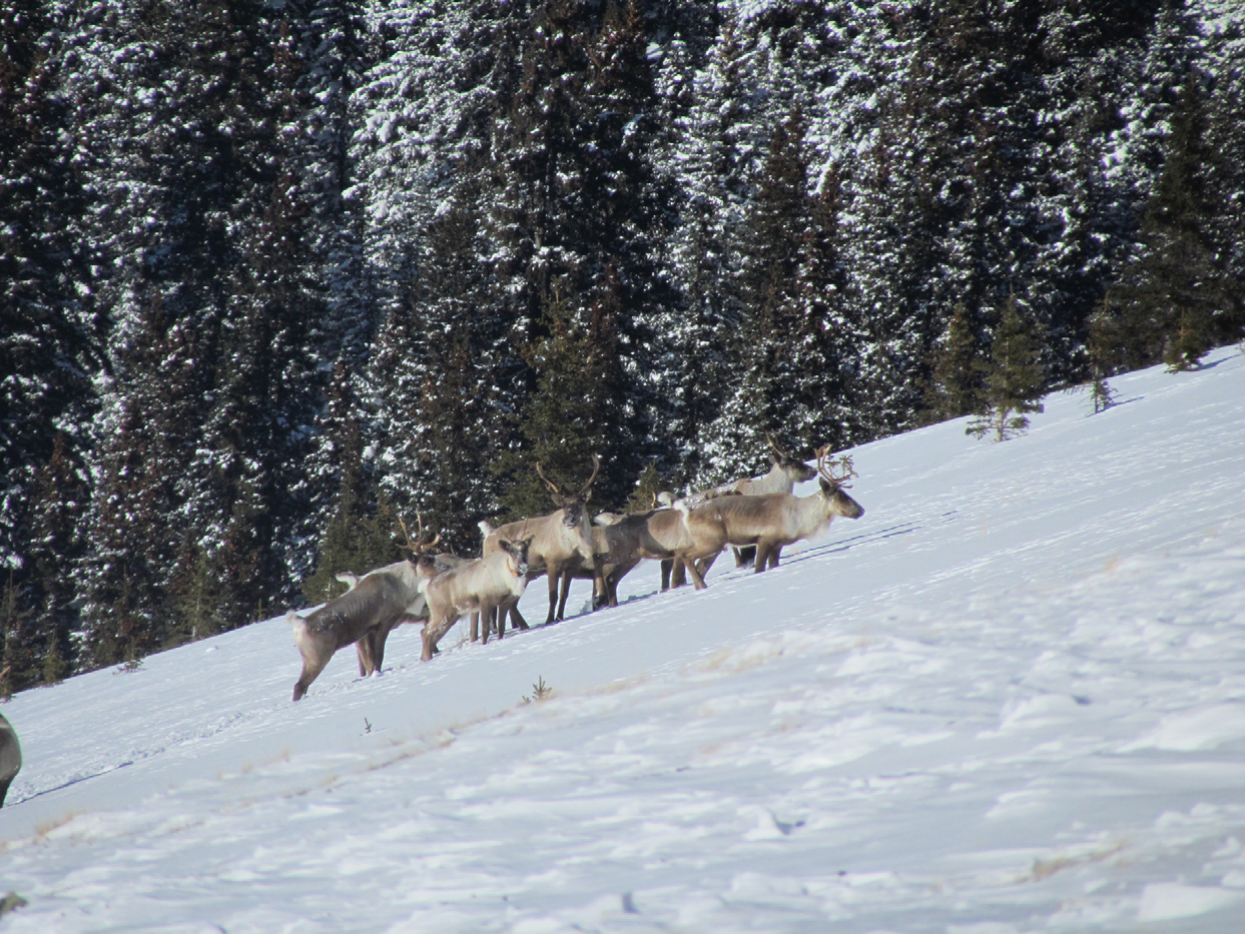
Photograph of central mountain woodland caribou.

**Figure 2:**
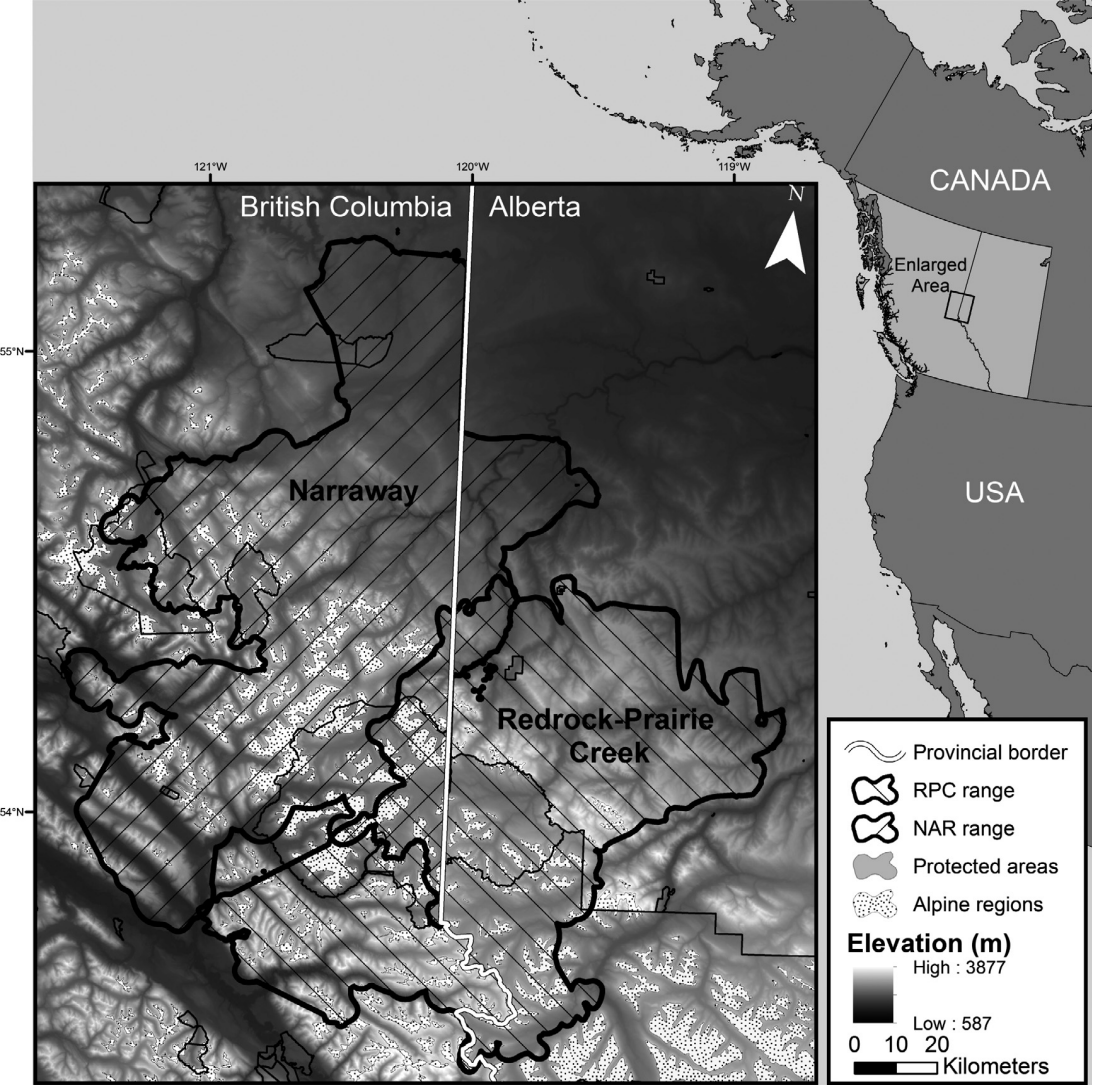
Redrock‐Prairie Creek (RPC) and Narraway (NAR) caribou ranges in west‐central Alberta and east‐central British Columbia, Canada, defined by provincial herd ranges and the 95% kernel distribution of GPS telemetry locations (1998–2013). The area where the two herd ranges overlap is crosshatched. Areas outside of protected areas are public lands.

The study area (17,325 km^2^) consisted of the Narraway and Redrock‐Prairie Creek caribou ranges along the continental divide between Alberta and British Columbia, Canada (Fig. 2). Narraway and Redrock‐Prairie Creek caribou are central mountain woodland caribou (Fig. 1) and migrate between high elevation summer range in alpine and subalpine habitat and low elevation winter range in the foothills (Edmonds 1988; Brown and Hobson 1998; COSEWIC 2014). Portions of Redrock‐Prairie Creek and Narraway ranges (35% and 28%, respectively) fall within parks and protected areas (Fig. 2). A detailed description of the flora and fauna in the study area is provided by DeCesare et al. (2012). Industrial development is concentrated in the foothills to the east of the continental divide. Oil and gas activities date to the 1950s and a coal mine has been operating in the eastern portion of the Redrock‐Prairie Creek range since 1969. Forestry operations date to the 1980s (Smith et al. 2000). In 2009, the Narraway and Redrock‐Prairie Creek populations were estimated at 100 and 212 individuals, respectively, and are listed as endangered by COSEWIC (2014).

### Telemetry data, seasons, and caribou UDs

Adult female caribou in the Redrock‐Prairie Creek (*n* = 93) and Narraway (*n* = 59) herds were captured between 1998 and 2013 using aerial netgunning and fitted with GPS telemetry collars (Lotek Engineering, Newmarket, Ontario, Canada; Appendix 1). Collaring was supervised by Alberta Environment and Parks under the Government of Alberta's Animal Care Protocol No. 008 (Hervieux et al. 2013). Because collaring took place in the fall when caribou aggregate in open areas for mating, spatiotemporal variation in year‐to‐year collaring effort was minimal and we assumed that individual caribou in the population had an equal chance of being collared and that our analysis was not biased by the collaring locations of individual caribou. We retained GPS telemetry locations for analysis if the recorded dilution of precision (DOP) was less than 10, resulting in 566,134 locations with a positional error of <35 m 95% of the time (Dussault et al. 2001; Appendix 1). To account for dynamics in the spatial distribution of caribou throughout the year, we defined seasons using an individual‐based recursive partitioning method that identifies seasonal onset dates (i.e., transition dates between seasons) based on inflection points in daily movement rates (Rudolph and Drapeau 2012). Methods for season delineation are detailed in Appendix 2.

We defined the total range of each herd as the combined extent of provincial herd ranges obtained from the governments of Alberta and British Columbia and the 95% kernel distribution of caribou telemetry locations (1998–2013). Provincial herd ranges in Alberta were created using historic caribou observations, telemetry data, and aerial surveys (Alberta Sustainable Resource Development & Alberta Conservation Association 2010), while British Columbia herd ranges were delineated using telemetry data and expert opinion (D. Seip, British Columbia Ministry of Environment, pers. comm.).

We estimated UDs for each individual/season/year combination. Collar failure, preprogrammed drop‐off, and mortality resulted in incomplete seasonal datasets for some individuals. We therefore only included individuals with telemetry data spanning the entire time frame of a given season. We calculated UDs using a fixed kernel method with a 250‐m cell size and the “plug‐in” method to determine the smoothing parameter *h,* such that *h* was optimized as a function of a normal distribution and the variance of the data in two dimensions (Sheather and Jones 1991). Kernel density estimates become more robust with large sample size; therefore, we used the complete telemetry dataset for individual UD estimation, and UDs were not estimated for individuals with <50 locations in a given season (Blundell et al. 2001). To account for biases stemming from unbalanced individual sample sizes and sampling intensities (fix rate; Appendix 1), we scaled individual UDs between 0 (zero probability of caribou use) and 1 (greatest probability of caribou use). We then created a population UD for each season, year, and herd by taking the average cell value of all scaled individual UDs per strata such that areas used intensely by many individuals had a higher value than areas used infrequently and by fewer individuals. To compare between strata, we scaled each population UD so that the sum of all cell values equaled 1. Population UDs per season, year, and herd were used in subsequent analyses. Because the distribution of telemetry locations forms the basis for the UD and accounts for the nonuniform intensity of use of available habitat throughout home ranges (Millspaugh et al. 2006), we used point data as a surrogate for UDs and extracted habitat variables (land cover, elevation) to evaluate the relative frequency of habitat use. We calculated UDs using the statistical software R (v3.1.1; R Development Core Team 2015) and the KernSmooth and raster packages (Hijmans 2014; Wand 2014).

To evaluate potential changes in caribou distribution over time, we built “baseline” UDs for each herd and season by merging individual UDs for caribou from 1998 to 2005. Obtaining accurate data on the historic distribution of wildlife is an obstacle for studies tracking distributional shifts over time (Tingley and Beissinger 2009), and methods for establishing baseline ranges for comparison to contemporary range depend on data availability, quality, and research objectives (see Faille et al. 2010; Clapp and Beck 2015; Turvey et al. 2015). Historic data on the distribution of Narraway and Redrock‐Prairie Creek caribou (i.e., Brown and Hobson 1998) lacked sufficient sample size to consider all seasons; thus, we built a baseline range using data from the initial phase of GPS data collection (1998–2005). We assumed this period (approximately one‐third of telemetry data) was sufficient to obtain a robust sample size to define a stable baseline range while accounting for small interannual variations in range use (Schaefer et al. 2000; Edwards et al. 2009). We did not assume that this baseline range corresponds with the historic distribution of caribou. Furthermore, we considered estimates of range shift in relation to this baseline range as conservative because range shift may have already commenced during the 1998–2005 period.

### Anthropogenic disturbance density, environmental and climatic covariates

We mapped the annual (1998–2013) disturbance footprint within Redrock‐Prairie Creek and Narraway caribou range for roads, oil and gas wells, and forestry clear‐cuts. Our primary interest was the response of caribou at the landscape scale; thus, we did not consider linear features such as pipelines and seismic lines that are generally not actively maintained and that caribou respond to at a finer scale (Dyer et al. 2001; DeCesare et al. 2012). We used spatial data from the Government of Alberta (Digital Integrated Dispositions for oil and gas wells and base road features), Weyerhaeuser Grande Prairie Co. Ltd (clear‐cuts and forest access roads), the Government of British Columbia (digital road atlas and forest harvest depletion layer; www.data.gov.bc.ca), and the British Columbia Oil and Gas Commission (well site point data; www.bcogc.ca/public-zone/gis-data). We verified the existence of disturbance features using annual SPOT imagery (SPOT 5‐7; www.blackbridge.com/geomatics). For well site point data from British Columbia, we applied a square buffer of 0.0158 km^2^, the average footprint of well sites in Alberta.

We calculated the cumulative density of disturbance features within each caribou range for each year (1998–2013) using a circular moving window average with a 1‐km radius; a conservative estimate of the influence of disturbance features on caribou at the landscape scale based on research showing that caribou respond to anthropogenic disturbance at distances up to 9 km (Schaefer and Mahoney 2007; Johnson et al. 2015). Disturbances occurring on the landscape before 1998 were included in the cumulative disturbance density. Cell values for disturbance density ranged between 0 (no disturbance) and 1 (completely disturbed) and represented the proportion of disturbed habitat within a 1‐km radius. We resampled disturbance density to a 250‐m cell resolution and scaled values so that the sum of all cell values equaled 1, analogous to the UDs estimated for caribou. We conducted density calculations in a geographic information system (GIS) using ArcMap 10.0 (Environmental Systems Research Institute 2015).

To assess trends in habitat use by caribou over time, we used the NASA Advanced Spaceborne Thermal Emission and Reflection Radiometer (ASTER) global Digital Elevation Model (DEM) with 30 m cell resolution (Tachikawa et al. 2011) as well as the spatial distribution of alpine habitat within caribou range (Alpine natural subregion in Alberta, Natural Regions Committee 2006; Boreal Altai Fescue Alpine biogeoclimatic zone in British Columbia; MacKenzie 2012). We also developed a 15‐class land‐cover classification (30 m resolution, see Nijland et al. 2015) from 2013 Landsat‐8 Operational Land Imager spectral data (Appendix 3). As our primary interest was caribou response to disturbance, we limited our analysis to two broad habitat categories that reflect the preference of caribou for herb and barren habitats (hereafter “nonforest”) during the summer, and conifer forest during the winter (Appendix 3; Brown and Hobson 1998; Johnson et al. 2015). We used the North Pacific Index (NPI; Trenberth and Hurrell 1994) to account for the influence of annual variation in climate on caribou distribution. NPI is a global climate index based on November to March sea surface pressure in the north Pacific that can affect climate with lag times of several months due to the mediation of climate effects through ocean–atmosphere circulation (Trenberth and Hurrell 1994; Lau et al. 2004). Hamel et al. (2009) previously correlated NPI to local weather patterns in our study area with high NPI values indicating relatively cold, snowy winters and low NPI values indicating relatively mild winters.

### Trends and relationship between caribou UDs, disturbance footprint, and environmental covariates over time

We used linear mixed models to quantify changes in climate severity (NPI) and the proportion of each individual's home range that was disturbed with respect to time. We defined home ranges by the 95% isopleth of individual kernel UDs and calculated home range size as the area within isopleths. We assessed changes in NPI over the study period (1998–2013) to account for variability in climate at the decadal scale (Trenberth and Hurrell 1994). As anthropogenic disturbance can influence caribou behavior and spatial distribution (e.g., Smith et al. 2000; Schaefer and Mahoney 2007), we assessed changes in home range size, daily movement rate (see Appendix 2 for methods), use of nonforest and conifer habitats, and average elevation of caribou locations in relation to time, the NPI, and the proportion of disturbed habitat within individual seasonal home ranges. We built models (R package lme4, Bates et al. 2014) for each season, herd, and dependent variable and specified the individual as a random effect to account for variation in behavior and landscape composition between individuals (Gillies et al. 2006). NPI was correlated with year (r̄ = 0.56); however, because pairwise variance inflation factors were <3 (Zuur et al. 2010), we included the NPI and year in models. Prior to analysis, we removed one large outlier in home range size that was more than double the size of all other home ranges.

We evaluated the relationship between population‐level caribou UDs, the disturbance density footprint, baseline caribou range, and alpine habitat using two indices of overlap; the probability that animal *i* would be found in animal *j*'s home range (PHR_*j,i*_) and the Utilization Distribution Overlap Index (UDOI; Fieberg and Kochanny 2005). PHR_*j,i*_ only requires a UD for animal *i* and is thus well suited for comparing UDs to a two‐dimensional feature such as alpine habitat for which a kernel density estimate is not appropriate. UDOI calculates the three‐dimensional product of two UDs and is a nondirectional index of overlap based on Hurlbert's (1978) *E*/*E*_uniform_ index of niche overlap (Fieberg and Kochanny 2005).

We used PHR_*j,i*_ to calculate the probability that population‐level caribou UDs would be located within (1) anthropogenic disturbance footprint, (2) baseline (1998–2005) seasonal range, and (3) alpine habitat for each herd, season, and year. We also calculated the UDOI between caribou UDs and (4) the anthropogenic disturbance footprint, and (5) baseline seasonal range for each herd, season, and year. We then examined trends in indices over time and in relation to NPI using multiple linear regression with PHR_*j,i*_ and UDOI as dependent variables. For the regression of UD overlap with baseline (1998–2005) seasonal range, we considered only caribou UDs collected between 2006 and 2013. We used R software (base and stats packages; R Development Core Team 2015) and code for PHR_*j,i*_ and UDOI from Fieberg (2014) for statistical calculations.

## Results

### Seasonal caribou UDs

Our dataset consisted of 329,668 locations from 93 Redrock‐Prairie Creek individuals and 236,466 locations from 59 Narraway individuals (Appendix 1). We identified six distinct caribou seasons: spring (Narraway May 5–June 1; Redrock‐Prairie Creek May 10–June 1), calving (June 1–June 20), summer (June 20–October 8), fall (October 8–November 29), early winter (November 29–February 5), and late winter (Narraway February 5–May 5; Redrock‐Prairie Creek February 5–May 10; Appendix 2).

**Figure 3:**
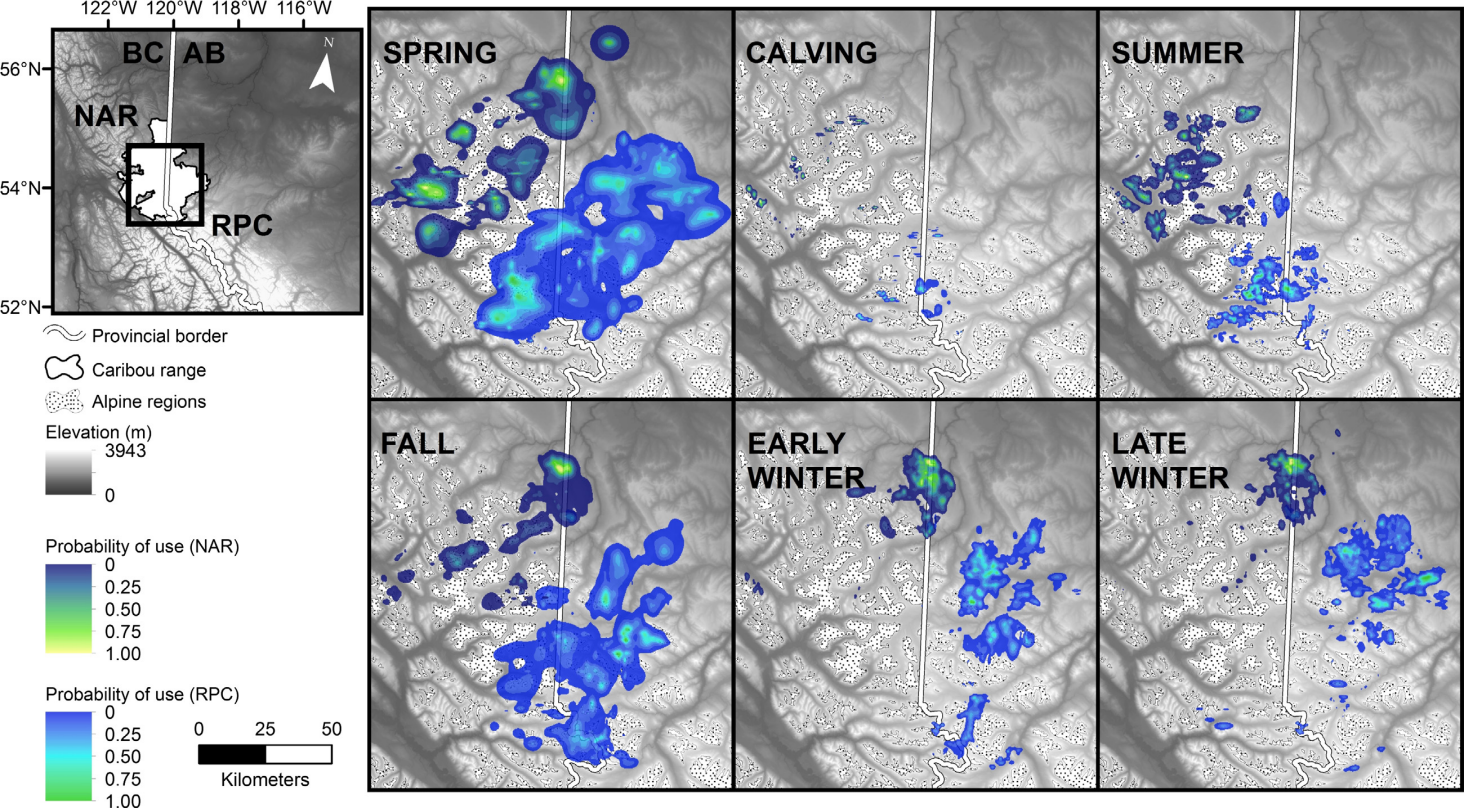
Baseline utilization distributions created using GPS telemetry data (1998–2005) for Redrock‐Prairie Creek (RPC) and Narraway (NAR) caribou. Panels show the relative probability of caribou use for six seasons in relation to elevation and alpine areas.

We estimated seasonal UDs for 152 individuals with an average of 6 animals per season per year (range: 1–17, SD: 2.83). Season/year/herd combinations with only one UD (*n* = 9) were removed from subsequent analyses. UD shape and placement on the landscape varied by season although both herds showed similar annual patterns (Fig. 3; Appendix 4).

### Anthropogenic disturbance density and climate

**Figure 4:**
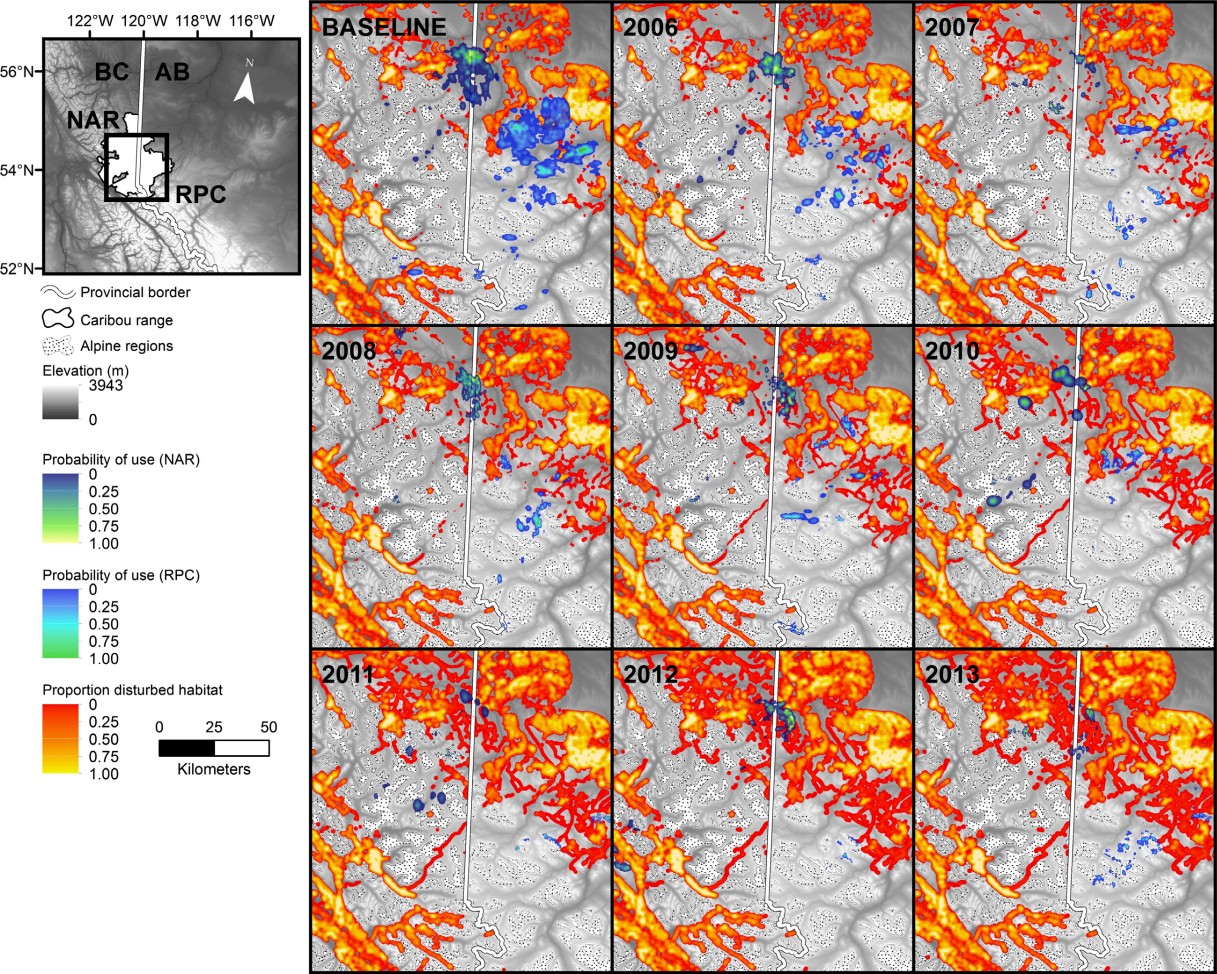
Baseline (1998–2005) and annual (2006–2013) UDs for Redrock‐Prairie Creek (RPC) and Narraway (NAR) caribou during late winter. Panels show the relative probability of caribou use and the density of anthropogenic disturbance features, elevation, and alpine areas.

Between 1998 and 2013, the area within Redrock‐Prairie Creek and Narraway range that was disturbed by clear‐cut logging, roads, and oil and gas well sites grew from 501 to 1177 km^2^. The proportion of caribou range <1 km from a disturbance feature increased from 10% to 35% (14–53% of unprotected land) for Redrock‐Prairie Creek caribou, and from 30 to 44% (42–61% of unprotected land) for Narraway caribou (Fig. 4; Appendix 5). Between 1998 and 2013, the disturbance footprint increased in both ranges (Narraway *P* < 0.001, *R* ^2^ = 0.98; Redrock‐Prairie Creek *P* < 0.001, *R* ^2^ = 0.69), but the proportion of disturbed habitat in each individual's home range remained stable (*P* = 0.51; *R* ^2^ < 0.001). NPI increased over the study period (*P* = 0.03; *R* ^2^ = 0.24).

### Trends in movement rate, home range size, and habitat use by caribou

Between 1998 and 2013, 95% home range sizes of Redrock‐Prairie Creek caribou decreased during early and late winter, and also decreased for Narraway caribou during late winter (Appendix 6). Over the study period, Redrock‐Prairie Creek caribou increasingly used high elevation during early winter, late winter, spring, and fall, while Narraway caribou increasingly used high elevation sites during early winter and spring (Appendix 6). Use of nonforest habitat increased over the study period during early winter, late winter, and spring for Redrock‐Prairie Creek caribou, and during spring for Narraway caribou (Appendix 6). Use of conifer habitat decreased over time during late winter and spring for Redrock‐Prairie Creek caribou, and during late winter for Narraway caribou (Appendix 6).

Between 1998 and 2013, home range size increased relative to the proportion of disturbed habitat within individual home ranges during summer and fall for Redrock‐Prairie Creek caribou, and during early winter, late winter, and fall for Narraway caribou (Table 1). Daily movement rate also increased in relation to anthropogenic disturbance during late winter, summer, and fall for Redrock‐Prairie Creek caribou and during late winter, calving, and summer for Narraway caribou (Table 1). Caribou home ranges with a greater proportion of anthropogenic disturbance occurred at lower elevations during early winter, late winter, summer, fall for Redrock‐Prairie Creek caribou, and during all seasons for Narraway caribou (Table 1). Use of conifer habitat increased relative to the proportion of disturbance within individual home ranges during calving, summer, and fall for Redrock‐Prairie Creek caribou, and during early winter and late winter for Narraway caribou (Table 1). Use of nonforest habitat decreased as disturbance increased during late winter, calving, summer, and fall for Redrock‐Prairie Creek caribou, and during all seasons except calving for Narraway caribou (Table 1). Increasing NPI was associated with a decrease in movement rate during early winter and late winter for Redrock‐Prairie Creek caribou, and with a decrease in elevation during calving for Narraway caribou (Appendix 6).

**Table 1:** Linear mixed effect model *β* coefficients (and standard errors) for the change in individual movement rate, home range size, and use of elevation, conifer, and nonforest habitat with respect to the proportion of disturbance within each individual home range for caribou in the Narraway and Redrock‐Prairie Creek ranges during six seasons between 1998 and 2013. Parameters with 95% confidence intervals that do not overlap zero are in bold

Herd	Variable	Season					
		Early winter	Late winter	Spring	Calving	Summer	Fall
Narraway	Movement rate (km/day)	−0.05 (0.04)	**0.13 (0.04)**	0.05 (0.10)	**0.19 (0.08)**	**0.15 (0.06)**	0.03 (0.08)
	Home range size (km^2^)	**21.53 (7.68**)	**14.62 (4.63)**	31.46 (23.81)	2.30 (1.87)	9.69 (6.58)	**37.62 (16.16)**
	Elevation (m)	**−44.88 (21.51)**	**−100.39 (26.67)**	**−62.05 (19.16)**	**−74.43 (24.96)**	**−29.33 (8.76)**	**−53.87 (17.58)**
	Proportion conifer	**0.07 (0.02)**	**0.09 (0.03)**	0.003 (0.03)	−0.01 (0.05)	0.01 (0.02)	−0.04 (0.03)
	Proportion nonforest	**−0.06 (0.026)**	**−0.10 (0.03)**	**−0.05 (0.02)**	−0.04 (0.06)	**−0.05 (0.02)**	**−0.042 (0.021)**
Redrock‐Prairie Creek	Movement rate (km/day)	0.06 (0.04)	**0.06 (0.027)**	0.11 (0.09)	0.15 (0.18)	**0.12 (0.055)**	**0.25 (0.05)**
	Home range size (km^2^)	8.64 (5.66)	6.86 (4.40)	−1.63 (25.33)	16.23 (18.75)	**21.74 (6.78)**	**61.30 (18.60)**
	Elevation (m)	**−40.88 (17.19)**	**−62.57 (19.84)**	−19.04 (20.56)	−54.97 (43.17)	**−28.07 (11.86)**	**−48.57 (9.99)**
	Proportion conifer	0.03 (0.03)	0.04 (0.03)	0.008 (0.03)	**0.16 (0.05)**	**0.08 (0.03)**	**0.046 (0.017)**
	Proportion nonforest	−0.06 (0.03)	**−0.07 (0.03)**	−0.04 (0.03)	**−0.15 (0.07)**	**−0.08 (0.03)**	**−0.07 (0.02)**

### Relationship between caribou UDs, disturbance density, alpine habitat, and environmental conditions over time

Over the study period, the use of alpine habitat (PHR_*j,i*_) by Redrock‐Prairie Creek caribou increased during spring, fall, and late winter (Table 2). Overlap between Redrock‐Prairie Creek caribou UDs and baseline range (UDOI) decreased for all seasons except calving and early winter (Table 2). Overlap between Redrock‐Prairie Creek caribou UDs and the disturbance footprint decreased during fall, but was stable for other seasons (Table 2). In relation to NPI, relatively cold, snowy winters were associated with an increased overlap the following fall between Redrock‐Prairie Creek caribou UDs and disturbance (Appendix 7). Relatively cold, snowy winters were also associated with a decreased probability of Redrock‐Prairie Creek caribou using areas within baseline range the following summer (Appendix 7).

**Table 2:** Multiple linear regression *β* coefficients (and standard error) for the change in the probability of caribou overlap with alpine habitat, disturbance footprint, and baseline (1998–2005) caribou distribution (PHR_*j,i*_), and the change in overlap (UDOI) between caribou utilization distributions, disturbance footprint, and baseline distribution over time (1998–2013) for caribou in the Narraway and Redrock‐Prairie Creek ranges during six seasons. Coefficients with 95% confidence intervals that do not overlap zero are in bold

Herd	Caribou UD Overlap with:	Season					
		Early winter	Late winter	Spring	Calving	Summer	Fall
Narraway	Alpine (PHR_*j,i*_)	0.01 (0.01)	0.02 (0.01)	**0.024 (0.007)**	0.02 (0.03)	−0.01 (0.01)	−0.004 (0.004)
	Disturbance (PHR_*j,i*_)	0.02 (0.02)	−0.01 (0.02)	**−0.02 (0.01)**	–	0.001 (0.01)	0.01 (0.01)
	Disturbance (UDOI)	**0.002 (0.00007)**	0.00001 (0.00007)	**−0.0008 (0.0003)**	–	0.00006 (0.00003)	0.0002 (0.0003)
	Baseline (PHR_*j,i*_)	−0.03 (0.01)	**−0.04 (0.01)**	0.002 (0.003)	−0.12 (0.05)	−0.01 (0.02)	0.01 (0.02)
	Baseline (UDOI)	**−0.05 (0.02)**	−0.03 (0.02)	**−0.021 (0.007)**	−0.02 (0.02)	−0.01 (0.01)	0.01 (0.02)
Redrock‐Prairie Creek	Alpine (PHR_*j,i*_)	0.01 (0.01)	**0.03 (0.01)**	**0.019 (0.008)**	0.06 (0.04)	−0.01 (0.01)	**0.013 (0.006)**
	Disturbance (PHR_*j,i*_)	0.0009 (0.01)	−0.02 (0.01)	−0.01 (0.01)	−0.02 (0.02)	0.0005 (0.01)	**−0.016 (0.006)**
	Disturbance (UDOI)	−0.00003 (0.00005)	0.0002 (0.00009)	−0.0002 (0.0003)	−0.00008 (0.0002)	0.000005 (0.00002)	**−0.00043 (0.00017)**
	Baseline (PHR_*j,i*_)	−0.01 (0.01)	−0.01 (0.01)	−0.004 (0.003)	−0.07 (0.08)	0.01 (0.01)	0.001 (0.01)
	Baseline (UDOI)	−0.03 (0.01)	**−0.04 (0.01)**	**−0.06 (0.01)**	−0.03 (0.04)	**−0.023 (0.009)**	**−0.04 (0.01)**

For Narraway caribou, the use of alpine habitat increased during spring but was otherwise stable, while overlap between Narraway caribou UDs and baseline range decreased during spring, early winter, and late winter (Table 2). Overlap between Narraway caribou UDs and the disturbance footprint decreased during spring and increased during early winter, but remained stable for other seasons (Table 2). For Narraway caribou, relatively cold, snowy winters were associated with an increase in overlap with disturbed areas and baseline range, a decrease in overlap with alpine habitats during the following spring, and an increased probability of using areas within baseline range during the following fall (Appendix 7).

## Discussion

Despite a significant increase in anthropogenic disturbance between 1998 and 2013 accounting for 14% and 25% of unprotected lands within the Narraway and Redrock‐Prairie Creek ranges, respectively, we observed a low and consistent degree of overlap between caribou UDs and the disturbance footprint throughout the study period. In addition, for all seasons except calving, we found a decrease in overlap between caribou UDs and baseline range. This apparent range shift across the last decade coincided with an increase in overlap between caribou UDs and alpine habitat, and an increase in the average elevation and proportion of nonforest habitat used by caribou during winter. The proportion of disturbed habitat within individual caribou home ranges did not change over the study period. Our findings complement previous research documenting a negative spatial response by caribou to anthropogenic disturbance in the same area (Smith et al. 2000; DeCesare et al. 2012). Our approach, however, is the first to use spatially explicit UDs to quantify changes in distribution over time in relation to the anthropogenic disturbance footprint and climatic trend of the region.

Our findings indicate that at the population level, caribou adjusted their spatial distribution and shifted their seasonal ranges such that overlap with disturbed areas at the landscape scale was minimal. These observed range shifts have resulted in a decreased use of former parts of caribou range, coincident with recent research that has documented population declines in both of these herds (Hervieux et al. 2013; Johnson et al. 2015). Monitoring the spatial distribution of caribou offers insight into the threats facing declining caribou populations that are not easily observed from demographic trends alone. For example, shifting ranges away from anthropogenic disturbance could alleviate some of the proximate threats faced by these herds (predation and stress associated with disturbance; Bradshaw et al. 1998; Hebblewhite et al. 2010), but the effectiveness of this strategy in contributing to the long‐term persistence of caribou likely depends on the availability and quality of alternative ranges (Saher and Schmiegelow 2005; Sawyer et al. 2006). If disturbance levels increase and caribou ranges contract further (i.e., Smith et al. 2000; Vors et al. 2007), the availability of alternative ranges that can provide adequate resources to maintain self‐sustaining populations may decrease (Saher and Schmiegelow 2005; Sawyer et al. 2006; Tracz et al. 2010). In this context, we believe that UDs are a simple and informative tool to monitor the distribution of caribou in relation to dynamics in spatially explicit landscape variables, and can contribute to conservation planning to increase the effectiveness and evaluate the success of recovery actions over time.

The change in distribution that we observed in these herds may be driven by several nonexclusive ecological processes. Predation risk for caribou is believed to increase as a function of anthropogenic disturbance (Courtois et al. 2007), and central mountain caribou may be able to reduce predation risk by shifting their distribution toward less disturbed portions of their range such as alpine habitats (Hebblewhite et al. 2010). We quantified an increase in the use of alpine habitat at the population level and a decrease in individual home range size over time, especially during winter, suggesting that caribou altered resource use, potentially to reduce their use of areas disturbed by anthropogenic activities (Edmonds 1988; Hebblewhite et al. 2010; Beauchesne et al. 2014). This strategy could prolong the persistence of caribou in the short term; however, the long‐term effects of increased use of high elevation areas are poorly understood and could present more complex challenges to the persistence of caribou (Barten et al. 2001; Sawyer et al. 2006; Schindler et al. 2007), and a decrease in individual home range size could indicate that caribou are confined to small pockets of suitable habitat that may become ecological traps (Beauchesne et al. 2014). Alpine environments have harsher weather, shorter growing season, and are less productive than low elevation sites (Barten et al. 2001; Natural Regions Committee 2006), and an increase in the use of alpine habitat during winter could potentially lead to greater thermoregulatory costs, poorer body condition, reduced reproductive success, reduced resistance to disease, and increased mortality (Crete and Huot 1993; Halvorsen et al. 1999). Additionally, while caribou may be able to reduce encounters with wolves by avoiding disturbed areas (Courtois et al. 2007), other predators such as cougars (*Puma concolor*), bears (*Ursus arctos* and *U. americanus*), and wolverines (*Gulo gulo*) could play a larger role in mortalities if caribou increasingly spend time in habitats where these predators are present (Kinley and Apps 2001; Pinard et al. 2012). Finally, increased use of alpine areas may expose caribou to increased mortality risk from stochastic weather events (i.e., extirpation of Banff caribou via avalanche [Hebblewhite et al. 2010; Johnson et al. 2015]). Because we monitored individual caribou for periods no longer than 2 years, we could not distinguish distributional shifts due to increased mortality in disturbed areas (i.e., Courtois et al. 2007) from distributional shifts due to behavioral adaptations of individuals to reduce predation risk. Collaring of the same individuals over a longer time period could provide additional insights into the mechanisms behind the observed distributional shifts.

Density‐dependent shifts in habitat use have been observed in large migratory caribou populations (Ferguson et al. 1998; Newton et al. 2015; Mahoney et al. 2016). Similarly, because recent mountain caribou population declines may have allowed a greater proportion of the caribou herd to make use of alpine habitat, density dependence could also explain the shifts in distribution that we observed toward greater use of alpine during winter. However, where density‐dependent effects have been observed, caribou populations are one to three orders of magnitude larger than those studied here and are believed to be primarily regulated by bottom‐up processes (Ferguson et al. 1998; COSEWIC 2014; Mahoney et al. 2016). In contrast, the literature suggests that mountain caribou are primarily regulated by high predation rates as opposed to forage limitations (Wittmer et al. 2005; Courtois and Ouellet 2007; Hervieux et al. 2014), although Wittmer et al. (2006) also provided some evidence for an effect of forage quality on mountain caribou survival and population dynamics that may be additive to the effects of predator‐mediated mortality on caribou. Therefore, while our data do not necessarily preclude density dependence as a causal mechanism driving range shifts in central mountain populations, we believe that there is currently little evidence to support this bottom‐up hypothesis as a primary driver of range shifts in our study area.

We observed a different pattern of range shift in the Narraway population than in the Redrock‐Prairie Creek population that may be explained by a landscape configuration effect, and by a response to predation at the individual and population level. The trend toward an increased use of alpine habitat was more apparent for Redrock‐Prairie Creek than for Narraway caribou, while the overlap with baseline ranges decreased at a similar rate for both herds. The discrepancy was not explained by differences in the proportion of alpine habitat (12% of Redrock‐Prairie Creek range; 11% of Narraway range), nor by the level of disturbance in each range, because while disturbance occurred in the Redrock‐Prairie Creek range at almost double the rate of the Narraway range, the total proportion of disturbed habitat within Narraway range (44%) was greater than Redrock‐Prairie Creek range (35%). It is possible that increased mortality of individuals and perceived predation risk in newly disturbed portions of caribou range may result in a restricted distribution due to the eventual disuse of those parts of the range (Smith et al. 2000; Vors et al. 2007; Briand et al. 2009). Thus, we would expect a positive relationship between the mortality rate and a shift away from baseline range toward alpine habitat that may be safer. However, the estimated population growth rates reported by Hervieux et al. (2013) during the study period for these populations were similar (95% CI 0.828–0.936 and 0.827–0.982 for Redrock‐Prairie Creek and Narraway, respectively), and thus, differential rates of decline do not explain the different patterns observed in each herd. We were unable to distinguish shifts in distribution due to perceived and realized predation risk from differences between herds in the availability and configuration of habitat types that are known to influence how caribou use habitat in a given range (Hins et al. 2009; Lesmerises et al. 2013); thus, we consider the availability of alternative range and changes in the spatial distribution of mortality risk as nonexclusive explanations for the differences in range shift observed between Redrock‐Prairie Creek and Narraway caribou, and additional research is necessary to tease out the relative contribution of each in determining the distribution of caribou.

NPI did not explain the decrease in overlap between caribou UDs and baseline caribou ranges. However, during relatively cold, snowy winters, we observed decreased movement rates, followed by increased overlap with disturbance during spring for Narraway caribou and during fall for Redrock‐Prairie Creek caribou, and a reduction in use of alpine habitat during spring for Redrock‐Prairie Creek caribou. Therefore, our results suggest that climate was not a significant factor in the distributional shifts observed for Redrock‐Prairie Creek and Narraway caribou, but did play a role in the ability of caribou to move away from disturbance. By limiting caribou movement rates and altering the future use of habitat, annual fluctuations in climate could limit the effectiveness of the moving‐away strategy for mountain caribou and may also change the viability of alternative ranges for caribou, especially as food availability and energetic costs in high elevation habitat are dependent on snow depth (Bradshaw et al. 1998; Johnson et al. 2001; Kinley et al. 2007).

During migratory spring and fall seasons, the increase in overlap between caribou distribution and disturbance over time could be due to the use of least‐cost migration corridors by caribou with low terrain ruggedness that are also preferred places for road construction (Saher and Schmiegelow 2005). Anthropogenic activity has been shown to increase potential for encounters with predators (Whittington et al. 2011). If anthropogenic disturbance and predation risk are concentrated in valleys used as migration corridors for caribou (i.e., Saher and Schmiegelow 2005; Whittington et al. 2005), the risk of migrating between summer and winter range could outweigh the benefits and we might expect to see changes in migratory patterns (i.e., partial migration or cessation of migration; Middleton et al. 2013) that would influence the seasonal distribution of caribou. Possibly, caribou currently mitigate some of the risks of migration by choosing alternate migration routes (i.e., ridgelines vs. drainages; Saher and Schmiegelow 2005) or displaying partially migratory behavior (McDevitt et al. 2009). However, continued development within caribou migration corridors and increased annual fluctuations in climate could influence the viability of alternate migration strategies by contributing to trade‐offs between energetic costs, foraging opportunities, and predation risk (McDevitt et al. 2009; Middleton et al. 2013).

Caribou distributions have been shrinking over the past century (Vors et al. 2007). Because we defined baseline caribou range using data acquired from 1998 to 2005, we probably underestimated the predisturbance seasonal range of caribou in our study area. Telemetry datasets rarely predate anthropogenic disturbance but in future studies, traditional ecological knowledge could be used to delineate boundaries of historic seasonal ranges and refine estimates of how caribou distribution has changed over a longer time frame (Ferguson et al. 1998). In addition, although our analysis found changes in the distribution of caribou over time in relation to disturbance and climate, these trends were not present in all seasons and the strength of these trends varied by herd and season. Within our study area, disturbance was concentrated in early and late winter seasonal ranges, a possible explanation for why effects were stronger in winter. Vors et al. (2007) found a lag of two decades between habitat disturbance and associated caribou range shift, which may also explain the variation in the strength of the trend that we detected across seasons. It is also possible that the time frame covered within this study was insufficient to detect a complete shift in caribou range. Additional monitoring of these caribou herds into the future may help to clarify the trends we present in this study. Complementary research quantifying the quality and availability of a variety of habitat types and alternative ranges, and monitoring of caribou body condition and health will contribute further insight on the implications of range shift on long‐term persistence of caribou populations.

In conclusion, we quantified a spatially explicit relationship between caribou distribution and anthropogenic disturbance over time. We also documented a shift in caribou distribution away from earlier ranges in a relatively short period in conjunction with an increase in anthropogenic disturbance. The range shift that we observed indicates a reduction in the use by caribou of areas altered by anthropogenic activities, thus allowing caribou to maintain a low overlap with anthropogenic disturbance across seasons. However, caribou populations continued to decline throughout the study period and this decline could indicate that the habitat currently available to caribou lacks adequate resources or predator avoidance opportunities for population persistence. Our approach using spatially explicit changes in UD overlap to make empirical inferences regarding the distribution of caribou in response to anthropogenic disturbance, habitat characteristics, and climate is simple, applicable to a wide range of species, and can be easily adapted to many ecological scenarios. We recommend the use of UDs for future studies investigating the implications and potential causes of observed changes in space use for the conservation of a variety of wildlife populations.

## Data Accessibility

Woodland caribou are listed as a threatened species under federal legislation, and thus, the locations collected with GPS telemetry are considered confidential. Given that caribou are generally loyal to habitats of seasonal importance such as calving sites and migratory corridors, they are considered particularly vulnerable to hunting and other forms of anthropogenic disturbance. As such, making telemetry locations publicly available would pose a serious risk to federal and provincial caribou recovery efforts, and it is under this discretion that we do not provide the GPS telemetry data used in this manuscript.

## Conflict of Interest

None declared.

## Figures and Tables

**Figure A1:**
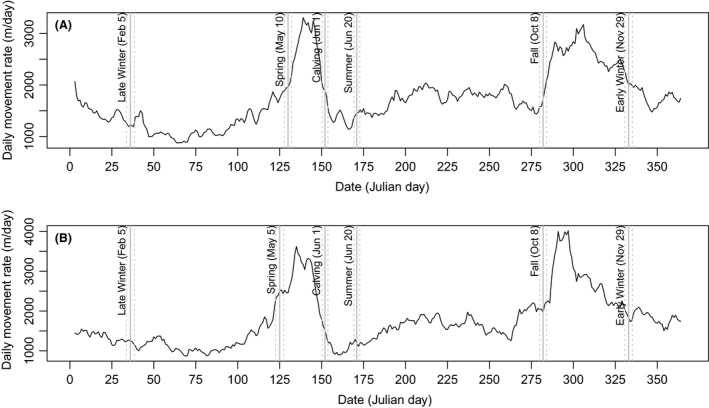
Seasonal onset dates and mean individual movement rates for caribou in the Redrock‐Prairie Creek (A) and Narraway (B) ranges between 1998 and 2013.

**Figure A2:**
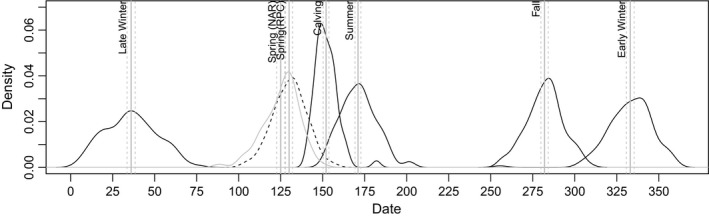
Density plot showing the distribution of seasonal onsets for individual caribou in the Redrock‐Prairie Creek and Narraway ranges from 1998 to 2013. Onset dates are pooled for both herds except for during spring; gray indicates spring onset for Narraway, while dashed black indicates spring onset for Redrock‐Prairie Creek. Gray vertical lines indicate mean onset dates (solid) and standard error (dashed) in Julian days.

**Figure A3:**
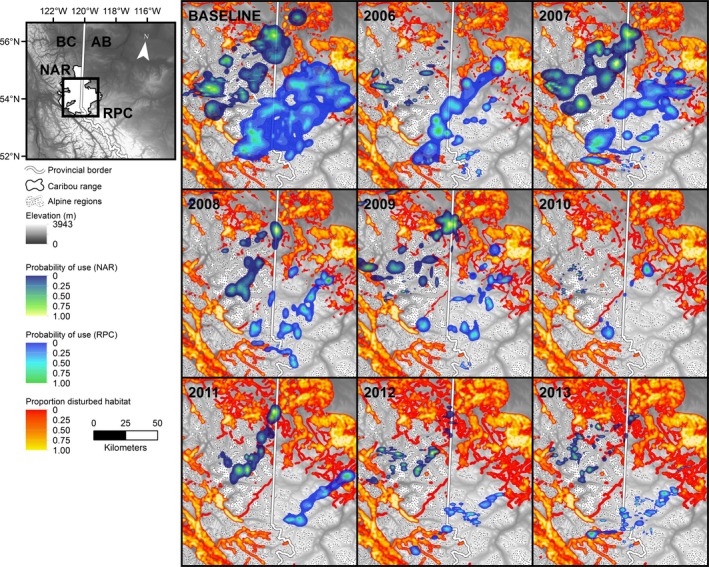
Baseline (1998–2005) and annual (2006–2013) UDs for Redrock‐Prairie Creek (RPC) and Narraway (NAR) caribou during spring. Panels show the relative probability of caribou use and the density of anthropogenic disturbance features, elevation, and alpine areas.

**Figure A4:**
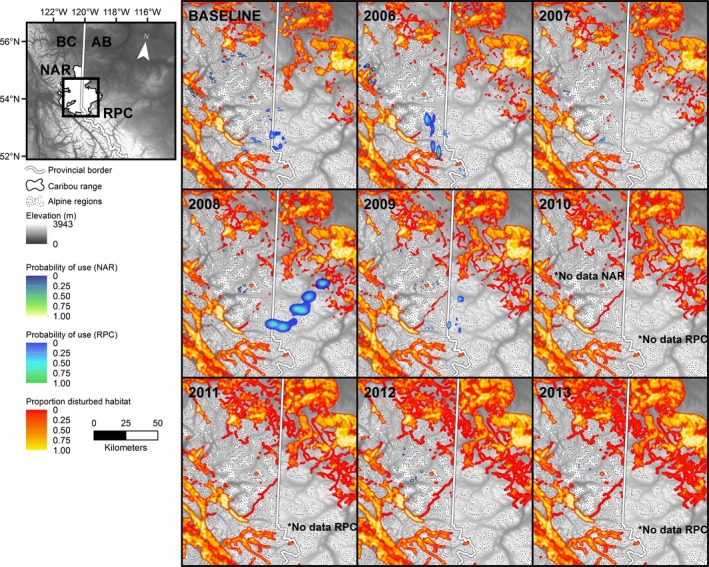
Baseline (1998–2005) and annual (2006–2013) UDs for Redrock‐Prairie Creek (RPC) and Narraway (NAR) caribou during calving. Panels show the relative probability of caribou use and the density of anthropogenic disturbance features, elevation, and alpine areas. There were insufficient data to calculate calving season UDs for Redrock‐Prairie Creek during 2010, 2011, and 2013, and for Narraway during 2010.

**Figure A5:**
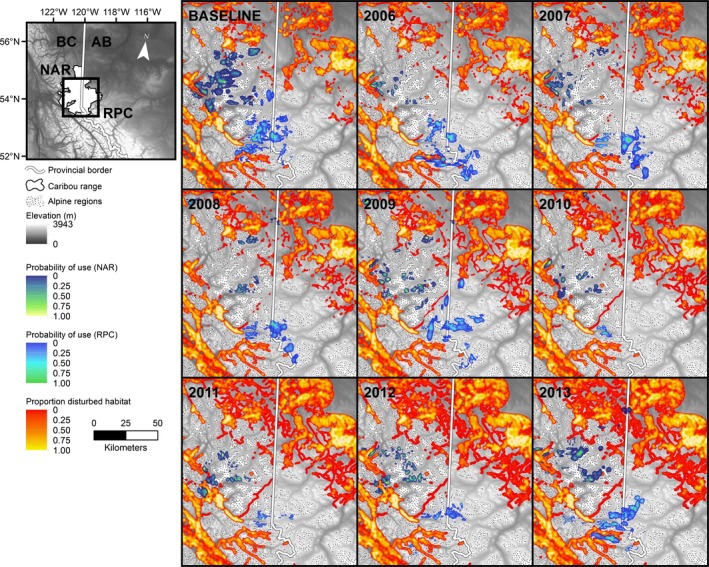
Baseline (1998–2005) and annual (2006–2013) UDs for Redrock‐Prairie Creek (RPC) and Narraway (NAR) caribou during summer. Panels show the relative probability of caribou use and the density of anthropogenic disturbance features, elevation, and alpine areas.

**Figure A6:**
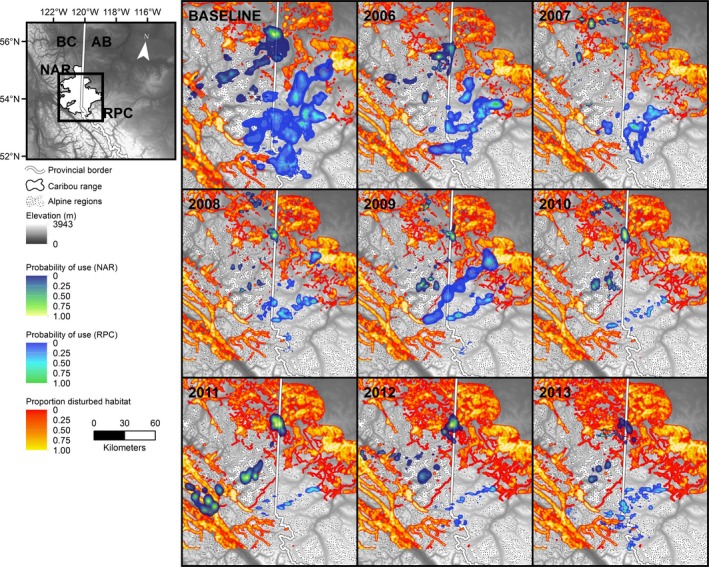
Baseline (1998–2005) and annual (2006–2013) UDs for Redrock‐Prairie Creek (RPC) and Narraway (NAR) caribou during fall. Panels show the relative probability of caribou use and the density of anthropogenic disturbance features, elevation, and alpine areas.

**Figure A7:**
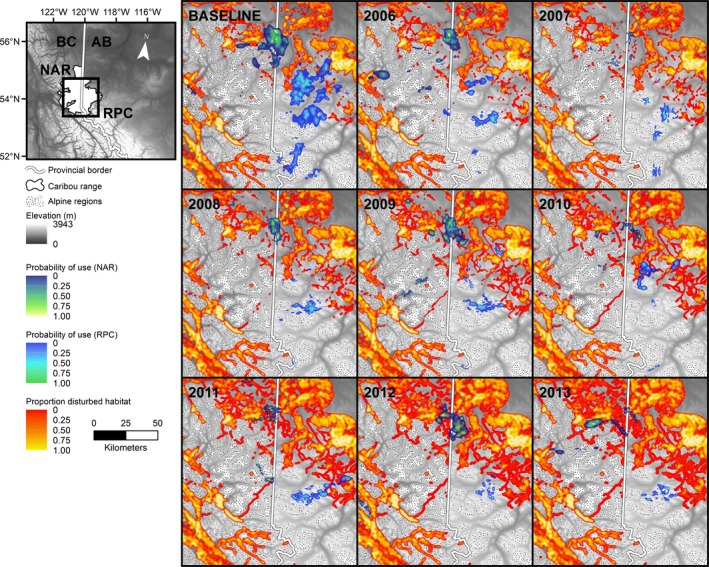
Baseline (1998–2005) and annual (2006–2013) UDs for Redrock‐Prairie Creek (RPC) and Narraway (NAR) caribou during early winter. Panels show the relative probability of caribou use and the density of anthropogenic disturbance features, elevation, and alpine areas.

## Appendix 1 Description of GPS telemetry data

### Methods

Adult female caribou in the Redrock‐Prairie Creek (*n* = 93) and Narraway (*n* = 59) herds were captured between 1998 and 2013 using aerial netgunning and fitted with GPS telemetry collars (Lotek GPS1000, 2000, 2200, 3300, 4400 models; Lotek Engineering, Newmarket, Ontario, Canada). Collaring was supervised by Alberta Environment and Parks under the Government of Alberta's Animal Care Protocol No. 008 (Hervieux et al. 2013). Over the monitoring period, the fix schedule varied by individual and time of year so that locations were collected at intervals of 1–6 h (average = 4 h). We considered GPS locations for analysis if the recorded dilution of precision (DOP) was less than 10, resulting in 566,134 locations (Table A1) with a positional error of <35 m 95% of the time (Dussault et al. 2001).

**Table A1:** Sample size of telemetry locations and collared individuals by year from Redrock‐Prairie Creek and Narraway caribou between 1998 and 2013. Raw data are all collected telemetry locations regardless of accuracy; cleaned data are those locations with a dilution of precision <10

Year	Redrock/Prairie Creek			Narraway		
	Telemetry locations			Telemetry locations		
	Raw	Cleaned	Individuals	Raw	Cleaned	Individuals
1998	2591	2054	5	–	–	–
1999	14,499	12,216	12	–	–	–
2000	45,443	41,579	24	3106	2643	5
2001	54,018	46,840	27	12,793	10,160	8
2002	19,883	15,499	18	21,414	17,274	12
2003	15,094	11,315	15	19,281	14,914	15
2004	11,679	9568	15	13,219	10,093	10
2005	17,909	15,709	18	11,302	10,362	12
2006	19,013	16,813	12	14,264	13,152	9
2007	18,939	17,805	15	17,549	16,083	14
2008	28,205	25,868	10	25,773	22,534	12
2009	25,951	23,863	7	26,822	24,400	7
2010	11,069	9638	8	13,299	12,318	8
2011	9486	9153	7	16,452	16,210	11
2012	20,656	20,480	6	29,183	28,841	8
2013	51,379	51,268	10	37,547	37,482	10
Total	365,814	329,668	93	262,004	236,466	59

## Appendix 2 Delineation of seasonal periods using recursive partitioning

### Methods

Recursive partitioning is an objective method to evaluate the modal pattern of movement rates while accounting for variation between individuals (Rudolph and Drapeau 2012). Movement patterns provide a biologically relevant link to seasonality, and transition periods are identifiable through temporarily elevated movement rates that indicate movement between seasonal ranges as opposed to smaller, within‐range movements (Ferguson and Elkie 2004). We calculated daily movement rates for each individual using a rarefied dataset (location closest to noon per day) to account for differences in fix rate between individuals. We calculated movement rates only when data from consecutive days were available, and only for individuals with >50 locations in a given year. We smoothed movement rates using a 5‐day moving window average to remove small fluctuations in movement rates that might interfere with the detection of larger movements between seasonal ranges (Basille et al. 2013). We determined onset dates for each individual and season based on inflection points in the daily movement rates, and we defined the population onset date for each season as the average of the individual onset dates around which individual onset dates were normally distributed. When no inflection point was clearly detected by recursive partitioning for a given individual and season, we excluded that individual from the calculation of the population onset date. Before calculating the population onset date, we used analysis of variance (ANOVA) to test for differences between individual onset dates by herd and year.

### Results

We identified six distinct seasons from inflection points in movement rates: spring (Narraway May 5–June 1; Redrock‐Prairie Creek May 10–June 1), calving (June 1–June 20), summer (June 20–October 8), fall (October 8–November 29), early winter (November 29–February 5), and late winter (Narraway February 5–May 5; Redrock‐Prairie Creek February 5–May 10) (Fig. A1). With the exception of spring, onset dates for seasons were normally distributed and ANOVA tests showed no difference between onset dates for herds or years; thus, we calculated a population‐level onset date for all seasons except spring as the average of all individual onset dates in both herds (Fig. A2). Individual onset dates for spring showed a bimodal distribution when we combined Narraway and Redrock‐Prairie Creek individuals, and an ANOVA test showed a significant difference between herds in the onset date for spring (*P* = 0.003). Therefore, we defined a herd‐specific onset date for spring as the average of individual onset dates within each herd.

## Appendix 3 Land‐cover classification for Narraway and Redrock‐Prairie Creek caribou range

### Methods

We developed a 15‐class land‐cover classification (30 m resolution) using available Light Detection and Ranging (LiDAR) data in part of the study area (adapted from Nijland et al. 2015) and applied on 2013 Landsat‐8 Operational Land Imager spectral data and the DEM (Table A2). To create annual land‐cover maps, we identified disturbance features from annual Landsat Thematic Mapper and Enhanced Thematic Mapper Plus images using a Vegetation Change Tracker approach (Huang et al. 2010) and then backfilled disturbed areas with the nearest undisturbed forest type. We combined classes into two broad habitat categories that reflect the preference of caribou for herb and barren habitats (“nonforest”) during the summer, and conifer forest during the winter (Table A2).

**Table A2:** Land‐cover categories, their descriptions, and reclassification for analysis of habitat use for Narraway and Redrock‐Prairie Creek caribou

Category	Description	Classification for analysis
Conifer Dense	>75% crown closure; >80% conifer based on stem count; “dry” or “mesic” moisture regime	Conifer
Conifer Moderate	40–75% crown closure; >80% conifer based on stem count; “dry” or “mesic” moisture regime	Conifer
Conifer Open	6–40% crown closure; >80% conifer based on stem count; “dry” or “mesic” moisture regime	Conifer
Herb	<25% shrub cover; <6% tree cover; “dry” or “mesic” moisture regime	Nonforest
Herb >2000 m	<25% shrub cover; <6% tree cover; elevation >2000 m	Nonforest
Bare	<6% vegetation cover	Nonforest
Bare >2000 m	<6% vegetation cover, elevation >2000 m	Nonforest
Mixed Closed	>50% crown closure; 26–79% broadleaf by count; “dry” or “mesic” moisture regime	N/A
Mixed Open	6–49% crown closure; 26–79% broadleaf by count; “dry” or “mesic” moisture regime	N/A
Deciduous Closed	>50% crown closure; >80% broadleaf based on stem count; “dry” or “mesic” moisture regime	N/A
Deciduous Open	6–49% crown closure; >80% broadleaf based on stem count; “dry” or “mesic” moisture regime	N/A
Wetland	>10% Vegetation Cover; “wet” or “aquatic” moisture regime	N/A
Agriculture	Designated for agricultural land use; crops, pasture etc.	N/A
Regeneration	>25% shrub or tree cover; Canopy <5 m height; “dry” or “mesic” moisture	N/A
Water	>6% standing or flowing water	N/A

## Appendix 4 Seasonal UDs for Narraway and Redrock‐Prairie Creek caribou in relation to movement patterns, habitat use, and home range size

Spring and fall UDs tended to be multimodal and larger in area than UDs in other seasons, reflecting the pattern of linear displacement punctuated by layovers in local areas displayed by central mountain caribou during migration periods (Table A3; Fig. 3). UDs for summer indicated that caribou spent the majority of that season in alpine environments (Table A3; Fig. 3). During calving, home ranges were small relative to other seasons; a reflection of the short duration of the season combined with low movement rates of calving females (Table A3). During fall, UDs shifted toward low elevation foothills on the eastern slope of the continental divide, and UDs remained at low elevation during early and late winter. During winter, caribou primarily used conifer forest habitat, whereas during calving and summer, caribou used a greater proportion of nonforest habitat (Table A3). Similar patterns were observed for both herds (Table A3; Fig. 3).

**Table A3:** Seasonal means (and standard errors) of individual daily movement rate, home range size (95% kernel home range), elevation, and proportion of locations in conifer and nonforest habitat for caribou in the Narraway and Redrock‐Prairie Creek ranges during six seasons between 1998 and 2013

Herd	Variable	Season					
		Early winter	Late winter	Spring	Calving	Summer	Fall
Narraway	Movement rate (km/day)	1.59 (0.03)	1.25 (0.02)	2.36 (0.08)	0.82 (0.07)	1.64 (0.03)	2.72 (0.07)
	Home range size (km^2^)	75.81 (11.54)	46.76 (8.19)	198.36 (29.43)	3.71 (0.96)	67.74 (8.77)	140.71 (21.87)
	Elevation (m)	1301.41 (16.65)	1344.90 (28.59)	1461.16 (20.96)	1713.82 (35.43)	1647.00 (13.87)	1379.65 (17.83)
	Proportion conifer	0.73 (0.02)	0.70 (0.04)	0.40 (0.03)	0.16 (0.04)	0.23 (0.03)	0.59 (0.02)
	Proportion nonforest	0.10 (0.02)	0.17 (0.03)	0.26 (0.02)	0.56 (0.06)	0.45 (0.02)	0.19 (0.02)
Redrock‐Prairie Creek	Movement rate (km/day)	1.63 (0.02)	1.21 (0.02)	2.29 (0.07)	1.17 (0.11)	1.72 (0.02)	2.56 (0.04)
	Home range size (km^2^)	50.24 (9.48)	28.46 (3.84)	172.90 (29.34)	41.50 (34.81)	85.10 (13.99)	202.88 (33.72)
	Elevation (m)	1712.40 (13.29)	1641.62 (23.64)	1626.35 (22.37)	1868.99 (43.95)	1805.12 (13.98)	1840.35 (10.78)
	Proportion conifer	0.57 (0.02)	0.66 (0.03)	0.58 (0.03)	0.25 (0.06)	0.33 (0.03)	0.31 (0.02)
	Proportion nonforest	0.37 (0.02)	0.27 (0.03)	0.28 (0.03)	0.62 (0.08)	0.60 (0.03)	0.63 (0.02)

## Appendix 6 Trends in caribou movement and habitat over time and in relation to anthropogenic disturbance density and climate

**Table A4:** Linear mixed effect model coefficients (and standard errors) for the change in individual movement rate, home range size, and use of elevation, conifer, and nonforest habitat with respect to time for caribou in the Narraway and Redrock‐Prairie Creek ranges during six seasons between 1998 and 2013. Parameters with 95% confidence intervals that do not overlap zero are in bold

Herd	Variable	Season					
		Early winter	Late winter	Spring	Calving	Summer	Fall
Narraway	Movement rate (km/day)	**−0.48 (0.02)**	−0.02 (0.01)	−0.004 (0.03)	−0.02 (0.03)	0.01 (0.02)	−0.02 (0.03)
	Home range size (km^2^)	−0.48 (2.63)	−2.54 (1.49)	−13.35 (7.99)	−0.18 (0.70)	0.91 (2.07)	−4.24 (4.60)
	Elevation (m)	**18.63 (8.06)**	16.22 (8.55)	**17.96 (6.47)**	12.87 (13.85)	1.46 (2.72)	2.84 (5.60)
	Proportion conifer	−0.01 (0.01)	**−0.03 (0.01)**	−0.01 (0.02)	−0.01 (0.02)	−0.01 (0.01)	−0.02 (0.01)
	Proportion nonforest	0.01 (0.01)	0.01 (0.01)	**0.021 (0.008)**	0.02 (0.03)	0.01 (0.01)	0.002 (0.01)
Redrock‐Prairie Creek	Movement rate (km/day)	−0.02 (0.01)	**−0.017 (0.008)**	−0.01 (0.03)	0.04 (0.09)	0.01 (0.01)	−0.01 (0.01)
	Home range size (km^2^)	−2.05 (1.62)	−**4.18 (1.20)**	−4.68 (6.92)	10.14 (10.20)	−0.21 (1.82)	−4.25 (5.07)
	Elevation (m)	**16.85 (5.26)**	**31.09 (5.56)**	**21.25 (5.64)**	29.94 (21.98)	−1.21 (3.26)	**5.82 (2.72)**
	Proportion conifer	−0.02 (0.01)	**−0.024 (0.008)**	**−0.016 (0.007)**	−0.02 (0.03)	−0.004 (0.01)	0.004 (0.005)
	Proportion nonforest	**0.022 (0.009)**	**0.03 (0.01)**	**0.021 (0.007)**	0.02 (0.04)	−0.003 (0.01)	−0.004 (0.01)

**Table A5:** Linear mixed effect model coefficients (and standard errors) for the change in individual movement rate, home range size, and use of elevation, conifer, and nonforest habitat with respect to the North Pacific Index for caribou in the Narraway and Redrock‐Prairie Creek ranges during six seasons between 1998 and 2013. Parameters with 95% confidence intervals that do not overlap zero are in bold

Herd	Variable	Season					
		Early winter	Late winter	Spring	Calving	Summer	Fall
Narraway	Movement rate (km/day)	−0.01 (0.02)	0.01 (0.02)	0.02 (0.06)	0.009 (0.05)	−0.02 (0.02)	−0.02 (0.04)
	Home range size (km^2^)	−4.26 (4.36)	−1.45 (2.62)	12.19 (12.84)	−0.24 (1.03)	−4.11 (3.42)	−6.15 (8.73)
	Elevation (m)	0.40 (10.58)	23.79 (14.92)	−12.80 (9.90)	**−25.12 (9.98)**	2.74 (3.59)	1.61 (9.20)
	Proportion conifer	−0.01 (0.01)	−0.02 (0.02)	0.02 (0.02)	0.01 (0.02)	−0.01 (0.01)	0.02 (0.01)
	Proportion nonforest	0.002 (0.01)	0.03 (0.02)	−0.02 (0.01)	−0.03 (0.03)	0.004 (0.01)	0.001 (0.01)
Redrock‐Prairie Creek	Movement rate (km/day)	**−0.07 (0.02)**	**−0.03 (0.01)**	−0.03 (0.06)	−0.12 (0.09)	−0.001 (0.02)	−0.03 (0.03)
	Home range size (km^2^)	−5.98 (3.64)	−2.39 (2.60)	−18.22 (16.33)	−8.72 (12.29)	7.66 (3.91)	7.02 (12.29)
	Elevation (m)	−4.60 (9.09)	−5.35 (11.16)	−0.98 (12.34)	−9.55 (24.20)	4.40 (4.51)	−1.05 (6.52)
	Proportion conifer	−0.001 (0.02)	−0.01 (0.02)	−0.01 (0.02)	−0.001 (0.03)	−0.004 (0.01)	−0.01 (0.01)
	Proportion nonforest	−0.005 (0.02)	0.02 (0.02)	0.01 (0.02)	0.02 (0.04)	0.01 (0.02)	0.01 (0.01)

## Appendix 7 Trends in caribou UD overlap with alpine habitat, baseline range (1998–2005), and anthropogenic disturbance over time and in relation to anthropogenic disturbance density and climate

**Table A6:** Multiple linear regression *β* coefficients (and standard error) for the change in the probability of caribou occurrence within alpine habitat, disturbance footprint, and baseline (1998–2005) caribou distribution (PHR_*j,i*_), and the change in overlap (UDOI) between caribou utilization distributions, disturbance footprint, and baseline (1998–2005) distribution with respect to the annual winter North Pacific Index for caribou in the Narraway and Redrock‐Prairie Creek ranges during six seasons between 1998 and 2013. Parameters with 95% confidence intervals that do not overlap zero are in bold

Herd	Caribou UD Overlap with:	Season					
		Early winter	Late winter	Spring	Calving	Summer	Fall
Narraway	Alpine (PHR_*j,i*_)	0.004 (0.03)	0.02 (0.01)	**−0.04 (0.01)**	0.02 (0.73)	0.01 (0.01)	−0.01 (0.01)
	Disturbance (PHR_*j,i*_)	−0.03 (0.03)	−0.01 (0.03)	0.04 (0.02)	–	−0.01 (0.01)	0.01 (0.02)
	Disturbance (UDOI)	0.00006 (0.0001)	0.0002 (0.0001)	**0.002 (0.0006)**	–	0.0001 (0.00005)	0.0007 (0.0006)
	Baseline (PHR_*j,i*_)	−0.02 (0.01)	0.01 (0.01)	0.01 (0.02)	0.15 (0.23)	0.04 (0.05)	**0.15 (0.05)**
	Baseline (UDOI)	0.01 (0.02)	−0.03 (0.02)	**0.06 (0.01)**	−0.05 (0.30)	0.01 (0.01)	−0.01 (0.03)
Redrock‐Prairie Creek	Alpine (PHR_*j,i*_)	−0.01 (0.01)	0.03 (0.01)	−0.003 (0.02)	−0.05 (0.46)	0.03 (0.01)	−0.02 (0.01)
	Disturbance (PHR_*j,i*_)	−0.03 (0.02)	−0.01 (0.02)	0.01 (0.01)	0.03 (0.39)	−0.001 (0.01)	**0.03 (0.01)**
	Disturbance (UDOI)	0.0001 (0.00009)	−0.00008 (0.0002)	−0.00008 (0.0004)	0.00002 (0.00003)	0.00001 (0.00003)	**0.001 (0.0003)**
	Baseline (PHR_*j,i*_)	−0.01 (0.02)	−0.05 (0.02)	−0.002 (0.005)	0.03 (0.84)	**−0.05 (0.02)**	−0.02 (0.01)
	Baseline (UDOI)	−0.01 (0.02)	−0.01 (0.02)	−0.01 (0.01)	−0.02 (0.79)	0.01 (0.01)	0.02 (0.01)
